# NCG 4.0: the network of cancer genes in the era of massive mutational screenings of cancer genomes

**DOI:** 10.1093/database/bau015

**Published:** 2014-03-07

**Authors:** Omer An, Vera Pendino, Matteo D’Antonio, Emanuele Ratti, Marco Gentilini, Francesca D. Ciccarelli

**Affiliations:** ^1^Department of Experimental Oncology, European Institute of Oncology, IFOM-IEO Campus, Via Adamello 16, 20139 Milan, Italy and ^2^Division of Cancer Studies, King’s College London, London SE1 1UL, UK

## Abstract

NCG 4.0 is the latest update of the Network of Cancer Genes, a web-based repository of systems-level properties of cancer genes. In its current version, the database collects information on 537 known (i.e. experimentally supported) and 1463 candidate (i.e. inferred using statistical methods) cancer genes. Candidate cancer genes derive from the manual revision of 67 original publications describing the mutational screening of 3460 human exomes and genomes in 23 different cancer types. For all 2000 cancer genes, duplicability, evolutionary origin, expression, functional annotation, interaction network with other human proteins and with microRNAs are reported. In addition to providing a substantial update of cancer-related information, NCG 4.0 also introduces two new features. The first is the annotation of possible false-positive cancer drivers, defined as candidate cancer genes inferred from large-scale screenings whose association with cancer is likely to be spurious. The second is the description of the systems-level properties of 64 human microRNAs that are causally involved in cancer progression (oncomiRs). Owing to the manual revision of all information, NCG 4.0 constitutes a complete and reliable resource on human coding and non-coding genes whose deregulation drives cancer onset and/or progression. NCG 4.0 can also be downloaded as a free application for Android smart phones.

**Database URL:** http://bio.ieo.eu/ncg/

## Introduction

Sequencing of exomes and genomes from thousands of cancer samples led to the identification of an increasing number of mutated genes that may contribute to driving human cancer (1–3). Owing to the massive amount of information derived from these studies, it is often difficult to get an overview of the genes that play a driver role in cancer on mutation (cancer genes). Since 2010, the Network of Cancer Genes (NCG) has been collecting information on a manually curated list of known and candidate cancer genes (4, 5). Known cancer genes have robust experimental support on their role in cancer onset and progression. Candidate cancer genes instead derive from large-scale mutational screenings of cancer samples and have been identified using statistical methods with poor or no experimental follow-up. Candidate cancer genes are thus prone to include false positives as a consequence of the difficult discrimination between passenger and driver mutations (6, 7). To account for this, NCG 4.0 reports a list of candidate cancer genes whose association with cancer is likely to be spurious owing to function, length and literature evidence.

For each known and candidate cancer gene, NCG 4.0 annotates a series of systems-level properties, defined as features that distinguish a group of genes (in this case, cancer-related genes) from the rest, and that cannot be ascribed to the function of the single gene alone (8). Systems-level properties currently reported in NCG are of evolutionary origin and duplicability, primary and secondary interaction network of the encoded proteins and miRNA regulatory networks. In addition, NCG 4.0 provides information on gene expression in 109 human tissues and on their functional characterization based on Gene Ontology (9). Owing to the increasing evidence of the primary role of microRNA (miRNA) deregulation in the onset of human cancer (10, 11), NCG 4.0 also annotates the systems-level properties of 64 cancer-related miRNAs (oncomiRs) manually derived from the literature.

**Figure 1.**
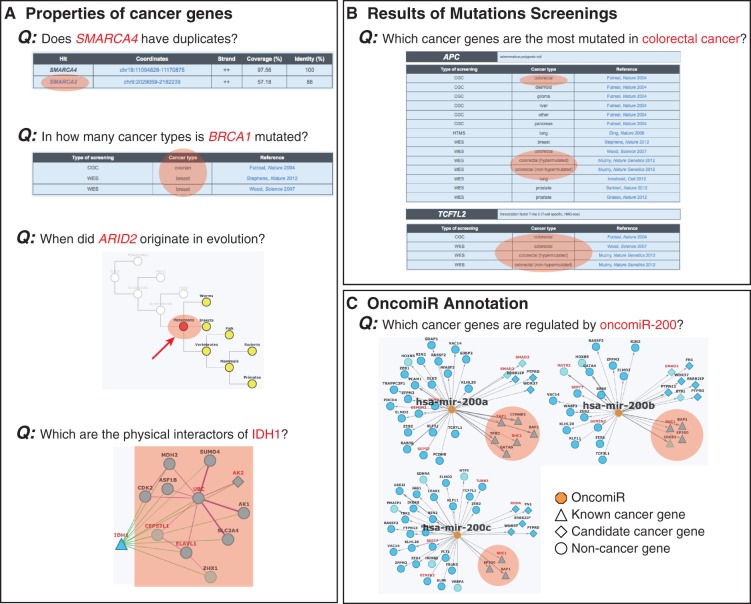
Examples of queries that can be done in NCG. Information stored in NCG can be used to address different queries regarding the properties of (**A**) individual cancer genes, (**B**) cancer types and (**C**) oncomiRs. Relevant information to address the specific queries is highlighted in orange.

Compared with other databases collecting all cancer mutations, such as COSMIC (12), ICGC (13) and CGAP (14), NCG 4.0 provides the community with a manually reviewed and constantly updated repository only of cancer drivers. In addition, it also annotates the properties of these genes, thus resulting useful to address different types of questions regarding cancer determinants (Figure 1) and to mine the increasing amount of information on cancer mutations.

## Database Description and Updates

### Manual collection of cancer genes

NCG 4.0 annotates the properties of 2000 cancer genes, defined as genes that contribute in promoting the onset and/or the development of human cancer. This list is derived from the union of two datasets. The first combined a literature-based repository of 484 genes from the Cancer Gene Census (377 dominant, 111 recessive and 4 genes that can act as both dominant and recessive, as frozen in January 2013) (15) with 77 genes whose amplification is causally implicated in cancer (16). This led to 537 experimentally supported cancer genes, which we defined as ‘known cancer genes’. The second dataset consisted of 1463 genes that are likely to be involved in cancer development on mutation, which we defined as ‘candidate cancer genes’. These genes derived from the manual revision of 67 publications corresponding to 77 re-sequencing screenings of the whole exomes (49 screenings), the whole genomes (19 screenings) and selected gene sets (9 screenings), conducted on 3640 samples from 23 cancer types (Supplementary Table S1) (17–83). These papers represented a comprehensive set of high-throughput cancer re-sequencing screenings.

**Figure 2.**
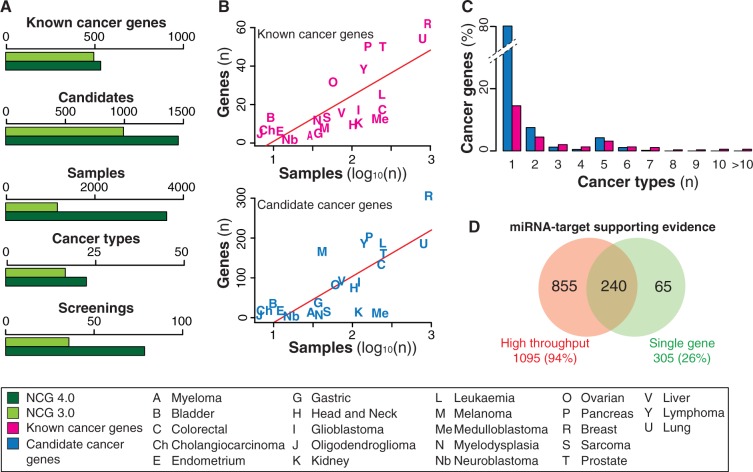
Overview of the data collected in NCG 4.0. (**A**) Comparison of data stored in NCG 3.0 and NCG 4.0. (**B**) Linear regression curves between the number of known and candidate cancer genes and the number of sequenced samples in each cancer type. Some cancer types deviate from linearity and this can be due to different reasons. For example, melanoma has a high number of candidate cancer genes (169) despite the low number of sequenced samples (41). In this case, the most likely explanation is that most of these candidate genes derive from two screenings (61, 75) that did not apply any methods to identify cancer drivers (Table 1, Supplementary Table S1). In the case of medulloblastoma, candidate and known cancer genes are only 25 despite 211 samples having been screened. This likely depends on the low mutation frequency of medulloblastoma [<1 mutation/Mb (40, 57, 64, 67)]. (**C**) Recurrence of known and candidate cancer genes in different cancer types. The only cancer genes that have been found mutated in more than 10 different cancer types are *TP53* (20 cancer types), *PIK3CA* (13 cancer types) and *PTEN* (12 cancer types). (**D**) Comparison of cancer miRNA targets that have been identified using single gene (i.e. reporter assay, western blot) and high throughput approaches (i.e. microarray, proteomic experiments and next-generation sequencing).

Compared with the previous version, NCG 4.0 appreciably increased the number of cancer genes, particularly candidates, and of sequenced samples (Figure 2A). Such accretion of knowledge reflects the current massive worldwide efforts to characterize cancer mutational landscapes in detail. Although we are expected to reach a plateau in the discovery of new driver genes because genes frequently (and significantly) mutated in some cancer types are also mutated at low frequency in other cancer types (1), our data show that we are still in the growing phase. In particular, for most cancer types the number of new candidate cancer genes increases with the number of sequenced samples (Figure 2B). As already noticed (1, 6), most cancer genes, and in particular candidates, are specific for a given cancer type, and only few known cancer genes recurrently mutate in several cancers (Figure 2C). This observation once again confirms the heterogeneity of cancer mutation landscape (3).

### Human gene set and orthology information

To identify the list of unique human genes, we aligned 33 427 protein sequences from RefSeq v.51 (84) to the reference human genome Hg19, using a method previously developed by our group (5, 8). This led to the identification of 19 045 unique gene loci, including 1961 of the 2000 cancer genes. Of the remaining 39 cancer genes, 29 did not have RefSeq protein entries and 10 were discarded because their protein sequences aligned to the genome for <60% of their length. For each cancer gene we retrieved duplicability, evolutionary origin, functional annotation, gene expression profile, protein–protein interaction and gene-microRNA interaction.

We assessed gene duplicability by the presence of one or more additional hits on the genome covering at least 60% of cancer protein length (8). Of the 1961 cancer genes, 325 (17%) had at least one extra copy on the genome. This was a significantly lower fraction compared with the rest of human genes (21%, *P*-value = 7.8 × 10^−^^06^, chi-square test), thus confirming the tendency of cancer genes to preserve a singleton status in the genome (8).

We assessed orthology relationships for 1978 of the 2000 cancer genes annotated in EggNOG v.3.0 (85) and used this information to infer the evolutionary origin of each cancer gene, defined as the most ancient node of the tree of life where the ortholog for that gene could be found (86). As already reported (86, 87), we confirmed that the fraction of old cancer genes that originated in prokaryotes and unicellular eukaryotes (1500, 76% of the total) was higher than in the rest of human genes (68%, *P*-value = 6.1 × 10^−^^13^, chi-square test). Moreover, we also confirmed that recessive cancer genes are older than dominant cancer genes (4). The vast majority of recessive cancer genes (87/111, 78%) originated already with the last universal common ancestor or with unicellular eukaryotes, compared with only 67% of dominant cancer genes (*P*-value = 0.03, chi-square test).

### Protein–protein and miRNA-target interaction networks

We rebuilt the human protein–protein interaction network integrating direct experimental evidence from five sources: HPRD (frozen on 13 April 2010) (88), BioGRID v.3.2.96 (89), IntAct v.159 (frozen on 14 December 2012) (90), MINT (frozen on 26 October 2012) (91) and DIP (frozen on 10 October 2010) (92). This resulted in a global network of 16 241 proteins (nodes) and 164 008 binary interactions (edges), supported by 33 497 independent publications. Interaction data were available for 1706 cancer proteins, and hubs (defined as proteins with at least 15 interactions) constituted 45% of all cancer genes, compared with 30% of the rest of human genes (*P*-value = 3.60 10^−^^38^, chi-square test).

The interaction network between miRNAs and cancer genes relied on experimental data extracted from three different sources: TarBase v.5.0 (93), miRecords v.4.0 (94) and miRTarBase v.4.4 (95). The integration of these data led to 1160 cancer targets of miRNAs (58% of the total). This was a significantly higher proportion compared with the rest of human genes (48%, *P*-value = 1.02 × 10^−^^17^, chi-square test) and confirmed the tendency of cancer genes to be regulated by miRNAs (4). This enrichment may reflect the fact that cancer genes are overall better characterized and thus more information is available on them. However, >70% of miRNA targets have been identified through high-throughput screenings (such as microarray, mass spectrometry and sequencing, Figure 2D), thus partially reducing the bias. Finally, we also updated the list of cancer genes that host miRNAs within their genomic loci (87 genes, 4.4% of the total).

## Novel Features of NCG 4.0

### Identification of possible false cancer genes

**Figure 3.**
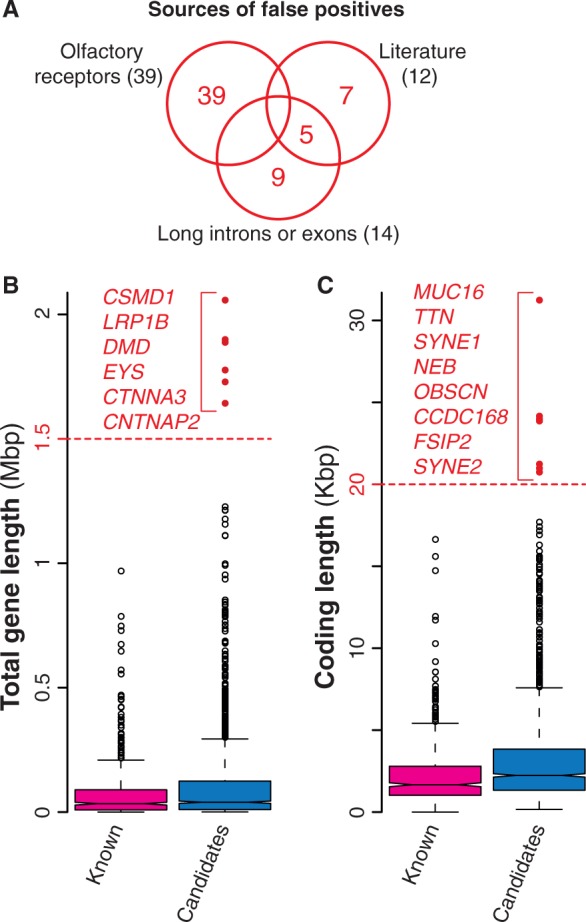
Possible false positives among candidate cancer drivers. (**A**) Venn diagram of the three groups of possible false positives. In total, we identified 60 genes, 65% of which were olfactory receptors, 23% were long genes and the remaining 20% were derived from literature (7). (**B**) Distribution of the total length for known and candidate cancer genes. Total gene length was measured as total number of nucleotides spanning the entire gene locus, including exons and introns. Red dots indicate possible false positives (gene longer than 1.5 Mb). (**C**) Length distribution of the coding regions for known and candidate cancer genes computed as the number of nucleotides covering the coding exons. Genes longer than 20 Kb (red dots) were considered as possible false positives.

With the increasing evidence of an overwhelming number of mutations acquired during cancer progression (most of which with no role in the disease), a number of statistical methods have been developed to identify cancer drivers within the whole set of mutated genes. These methods take into account several features including the tendency of the same gene to be mutated across many samples, the cancer-specific background mutation rate, the gene length and expression and the mutation effect on the encoded protein (Table 1, Supplementary Table S1). Despite all efforts to refine the identification of driver mutations, current approaches are still prone to false positives, i.e. mutated genes that are erroneously identified as cancer drivers (6, 7). For example, genes encoding olfactory receptors are often included in the list of candidates, because they tend to mutate although the biological function and expression pattern of these genes strongly dismiss a possible functional role in the disease. Similarly, overly long genes are also probable false positives because their recurrent mutations in several samples are most likely due to their length more than to their function (6, 7). Because the main goal of NCG is to annotate the properties of cancer genes, we decided to collect all putative cancer genes from primary data without removing possible false positives. However, we added a warning concerning the possible spurious cancer associations for 60 genes (39 olfactory receptors, 14 genes with long exons and/or introns and 7 additional false positives derived from literature (7) (Figure 3A, Table 1). Although gene length by itself does not imply spurious associations, we derived the length distributions of all candidate cancer genes and considered genes with long introns (Figure 3B) or long exons (Figure 3C) as possible false positives.

**Table 1.** Methods used to identify candidate cancer genes and possible false positives

Method	MuSiC (96)	Mutsig (7)	Wood *et al.* (80, 97)	Greenman *et al.* (98)	Paper-specific	Recurrence-based	None
Candidate cancer genes	Genes that mutate with significantly higher rate than the background, considering multiple mutational mechanisms. It allows for pathway and proximity analysis, clinical correlation test and PFAM/OMIM query	Genes that mutate more often than expected, given the background mutation rate. It clusters mutations in hotspots and considers the functional impact and the conservation of the genomic site. The latest version takes into account patient and genomic mutation patterns	Genes that (a) mutate in both discovery and validation screens; (b) whose mutations exceed a certain threshold and; (c) mutate at a frequency higher than the passenger mutation rate	Genes that mutate at higher frequency than expected. Expectation is estimated using silent mutations	*Ad hoc* methodology developed for the specific set of samples and cancer type analyzed in the paper	Recurrence of mutations in a gene within samples is taken as evidence of its causal involvement in disease onset. Particularly used when few samples and/or cancer types with low mutation instability are analyzed	Often associated to whole genome screening, when only one or very few samples are sequenced. In such cases, all mutated genes are retained as possible candidates
Number of screenings	5	17	13	3	10	17	12
Possible false positives	*6 (LRP1B, OR6A2, OR11L1, OR5B17, OR10G7, RYR2)*	*17 (CNTNAP2, CSMD3, LRP1B, ORC4C15, OR8H2, OR8K1, OR6K3, OR5L2, OR2T33, PCLO, LRP2, MUC4, NEB, RYR2, SYNE1, SYNE2, TTN)*	*9 (CCDC168, CNTNAP2, CSMD3, EYS, LRP2, MUC16, OR2L13, OR51E1, TTN)*	*None*	*15 (CSMD3, CNTNAP2, OR4L1, OR10G9, OR5L1, OR4K14, OR4C13, OR4C6, OR51L2, OR1M1, OR2A42, OR10AG1, OR2A2, OR4K1, OR52E8)*	*13 (CNTN5, CSMD3, DMD, LRP1B, FSIP2, OR2M4, OR10R2, OR1L8, OR4C46, PCLO, RYR2, SYNE1, TTN)*	*17 (CTNNA3, CNTN5, CSMD1, OR2T11, OR2T34, OR5M5P, OR4S2, OBSCN, OR1J2, OR4D11, OR5H2, OR4Q3, OR4N5, OR52A5, RYR3, SYNE2, TTN)*

For each method used to identify candidate cancer genes (i.e. new possible cancer drivers) in the 77 screenings, reported are a brief description of the procedure, the number of screenings that relied on it and the associated possible false positives.

### Gene expression profiles

To complete the functional annotation of cancer genes, we derived expression levels for 1528 of them from two high-throughput gene expression experiments on 109 human tissues (99, 100). We normalized and processed the raw CEL files obtained from the corresponding Gene Expression Omnibus series (GSE2361 and GSE1133) using the MAS5 algorithm of the R *affy* package (101, 102). Because more than one probe can be associated with one gene, the expression level of each cancer gene in a given tissue was defined as the mean expression levels of all probes with detection *P* < 0.05. If all probes for a gene had detection *P* > 0.05, the gene was considered as not expressed.

To make a comparative assessment of the expression levels of a cancer gene *i* in a given tissue *t* with those of all other genes in the same tissue, we first calculated the expression levels of all human genes in that tissue. We then derived the normalized expression level *n* of the cancer gene *i* in the tissue *t*, measured as:

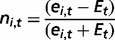

where *e_i__,__t_* was the expression level of the cancer gene *i* in tissue *t* and *E_t_* was the median expression level of all genes in tissue *t*. Normalized expression levels allowed a direct comparison of the expression of all genes in each given tissue.

### Manual collection of miRNAs involved in human cancer (oncomiRs)

We manually gathered the list of oncomiRs from the literature and included only miRNA families (i.e*.* miRNAs with high sequence similarity) and miRNA clusters (i.e. miRNAs that are neighbors in the genome and co-transcribed) whose role in cancer was well described and experimentally supported (103–108). This led to 64 oncomiRs involved in 27 cancer types. Similarly to protein-coding genes we retrieved details on duplicability, evolutionary origin and interaction network for all these oncomiRs.

To infer oncomiR duplicability, we downloaded 1424 human miRNAs from miRBase v.17 (109) and considered all mature miRNAs with the same seed (i.e. the 6–8 nt-long region at the 5′-end of the sequence) as duplicated miRNAs. The rationale for this choice was that, because seeds determine the specificity in target recognition, their sequences are the most conserved among homologous miRNAs (110). Among 64 oncomiRs, 51 (79%) were duplicated compared with 33% other duplicated human miRNAs (*P* = 4.5 × 10^−^^16^, chi-square test). Therefore, unlike protein-coding cancer genes that maintain a singleton status in the genome, oncomiRs tend to have additional copies that share the site of recognition of the RNA targets.

To pinpoint when oncomiRs appeared in evolution, we developed a procedure similar to that used for protein-coding genes and traced the most ancient miRNA ortholog. We first retrieved the orthologs of 835 human miRNAs for which miRNA families were available in miRBase (including all 64 oncomiRs). We then assigned the origin of each miRNA as the most ancient ortholog within the corresponding family. Sixty oncomiRs (94% of the total) had orthologs in vertebrates, compared with only 19% of the rest of human miRNAs, thus suggesting that oncomiRs originated earlier than the rest of human miRNAs. It is worth noticing that the marked differences in duplicability and origin between oncomiRs and other human miRNAs are at least partly inflated by the high interest in oncomiRs that boosted the search of their paralogs and orthologs in other species.

## Web Interface, Implementation and Data Availability

NCG 4.0 runs on an Apache web server and data are stored in a MySQL database. The web interface was developed in PHP and network visualization was implemented in Cytoscape Web (http://cytoscapeweb.cytoscape.org/) (111).

We modified NCG 4.0 web interface to enhance functionalities and facilitate the retrieval of the properties of cancer genes and oncomiRs. In addition to searching for single genes or list of genes of interest, the user can now visualize and browse all 2000 cancer genes, as well as retrieve cancer genes based on specific filters. NCG 4.0 also provides a detailed report on the cancer types and the corresponding publications where it was found mutated. Similar types of searches can be done on the 64 oncomiRs.

All data stored in NCG 4.0 are summarized in the statistics section that provides an overview on the properties of cancer genes and oncomiRs. For example, it is possible to compare mutation frequency, number of cancer genes and oncomiRs as well as their recurrence across the different cancer types and screenings. The bulk content of the database as well as the list of cancer genes, false positives and oncomiRs can be downloaded as text files. We developed a mobile phone application for NCG 4.0 that is freely available from the Web site.

## Supplementary Data

Supplementary data are available at *Database* online.
